# The Innate Immune Cross Talk between NK Cells and Eosinophils Is Regulated by the Interaction of Natural Cytotoxicity Receptors with Eosinophil Surface Ligands

**DOI:** 10.3389/fimmu.2017.00510

**Published:** 2017-04-28

**Authors:** Silvia Pesce, Fredrik B. Thoren, Claudia Cantoni, Carola Prato, Lorenzo Moretta, Alessandro Moretta, Emanuela Marcenaro

**Affiliations:** ^1^Dipartimento di Medicina Sperimentale, Università degli Studi di Genova, Genova, Italy; ^2^Sahlgrenska Cancer Center, University of Gothenburg, Göteborg, Sweden; ^3^Istituto Giannina Gaslini, Genova, Italy; ^4^Centro di Eccellenza per le Ricerche Biomediche, Università degli Studi di Genova, Genova, Italy; ^5^Department of Immunology, IRCCS Bambino Gesù Children’s Hospital, Rome, Italy

**Keywords:** NK cells, eosinophils, dendritic cells, natural cytotoxicity receptor, cross talk, innate cells, cytotoxicity

## Abstract

Previous studies suggested that the cross talk between NK cells and other cell types is crucial for the regulation of both innate and adaptive immune responses. In the present study, we analyzed the phenotypic and functional outcome of the interaction between resting or cytokine-activated NK cells and eosinophils derived from non-atopic donors. Our results provide the first evidence that a natural cytotoxicity receptor (NCR)/NCR ligand-dependent cross talk between NK cells and eosinophils may be important to upregulate the activation state and the effector function of cytokine-primed NK cells. This interaction also promotes the NK-mediated editing process of dendritic cells that influence the process of Th1 polarization. In turn, this cross talk also resulted in eosinophil activation and acquisition of the characteristic features of antigen-presenting cells. At higher NK/eosinophil ratios, cytokine-primed NK cells were found to kill eosinophils *via* NKp46 and NKp30, thus suggesting a potential immunoregulatory role for NK cells in dampening inflammatory responses involving eosinophils.

## Introduction

NK cell function is to a large extent regulated by activating and inhibitory cell surface receptors. A number of triggering receptors responsible for NK cell activation have been identified and molecularly characterized during the last decade. For example, NKp46, NKp30, and NKp44 (collectively termed natural cytotoxicity receptors, NCRs) are mostly expressed by NK cells and represent crucial receptors for the recognition and killing of most target cells (1–3). In recent years, two NKp30 ligands were identified, including the HLA-B associated transcript 3 protein (BAG6) and B7-H6 (4–6), but the identity of the endogenous cell surface ligands for NKp46 receptor remains mostly unknown (7). Other activating receptors involved in target cell recognition and lysis are represented by NKG2D, DNAM-1, 2B4, and NTBA (3). In contrast to NCRs, the cellular ligands for these receptors have been identified. NKG2D recognizes stress-induced ligands such as MICA/MICB and ULBPs, whereas DNAM-1 recognizes poliovirus receptor (PVR, CD155) and Nectin-2 (CD112); 2B4 recognizes CD48, while NTBA mediates homotypic interactions (8).

Human NK cells also express inhibitory receptors, comprising a variety of HLA class-I-specific ones that include killer cell immunoglobulin-like receptors and the CD94/NKG2A heterodimer (9–12), which, upon interactions with self-HLA class-I molecules, prevent NK cell-mediated attack of autologous healthy cells. On the other hand, cells in which HLA class-I expression is downregulated (for example, following tumor transformation or viral infection) become susceptible to NK-mediated killing.

In addition to their important role in the control of viral infections and malignancies, recent studies indicate that NK cells can efficiently participate to the shaping of adaptive immune responses (13–16). In this context, it has been shown that activated NK cells, by a mechanism termed “dendritic cells (DCs) editing,” may contribute to the quality control of DCs undergoing maturation by exerting a selection of the fittest DCs for optimal antigen presentation (17–19). Accumulating evidence suggests that the NK cell influence on the adaptive immune response is also tuned by other innate immune cells that are localized at the site of infection or in the tumor microenvironment (20, 21). These cells further modulate the ability of NK cells to regulate DC editing and maturation, either by releasing type I or type II cytokines or by directly interacting with NK cells (22). Thus, the effect of the interaction between NK and DCs may be conditioned by the characteristics of the inflammatory microenvironment in which immune responses occur (17, 23). Moreover, NK cells may deliver important signals contributing to T cell polarization toward type 1 (Th1) immune responses directly in secondary lymphoid compartments (SLCs) (13, 17, 24–26).

Eosinophils are an end-stage type of granulocyte derived from primordial stem cells in the bone marrow that is known to circulate through the peripheral bloodstream and tissues. After trafficking to tissues, eosinophils bind to specific sites because of an extracellular matrix protein, fibronectin.

Subsequently, eosinophils receive signals to degranulate and release the preformed components of their granules, such as major basic protein, eosinophil cationic protein, eosinophil-derived neurotoxin, and eosinophil peroxidase. These proteins target any foreign antigen, promote inflammation to the area, and may cause significant damage to surrounding structures (27, 28).

Moreover, eosinophils by releasing several type I and type II cytokines, growth factors, and chemokines can display both pro-inflammatory and anti-inflammatory activities (27, 28). Eosinophils express receptors for many of these soluble factors (that promote longevity of eosinophils in tissues), as well as innate receptors including pattern recognition receptors such as toll-like receptors (TLR 1–5, 7, 9) (29).

In addition, it was proposed that eosinophils may process and present a variety of microbial, viral, and parasitic antigens and, following activation, express high levels of HLA class-II and co-stimulatory molecules, and upregulate CD62L. For these reasons, eosinophils may rapidly traffic to regional lymph nodes, where they can function as antigen-presenting cells (APCs) promoting CD4^+^ T cell proliferation and polarization (30–35). Importantly, eosinophils are observed in the peritumoral infiltrate of several types of cancers (27, 36), and the presence of tumor-associated eosinophilia seems to correlate with a better tumor prognosis (37).

Here, we show that, following coculture and direct cell-to-cell contact with eosinophils, NK cells upregulate their effector function. This process is dependent on the engagement of NKp46 and NKp30 triggering receptors. The increase of NK cell-mediated IFNγ production and cytotoxic activity against tumor cells results in an increased ability of NK cells to perform an efficient editing of DCs. In addition, we show that eosinophils acquire an activated phenotype, by the *de novo* expression of CD69, ICAM-1, and HLA class-II molecules. Moreover, the upregulation of CD62L confers to eosinophils a migratory capacity to SLC of cells and the acquisition of features of APCs. Interestingly, at higher NK/eosinophil ratios, cytokine-primed NK cells exert cytotoxic activity toward eosinophils through the engagement of NKp46 and NKp30, thus exerting a possible control on eosinophil survival and activity during the late phases of inflammatory responses.

## Materials and Methods

### Monoclonal Antibodies

The following mAbs produced in our laboratory were used in this study: anti-HLA class-I (A6/136, IgM), anti-2B4 (CO54, IgM), anti-NTBA (MA127, IgM), anti-CD48 (CO202, IgM), anti-CD9 (M1B16 IgM), anti-DNAM-1 (F5, IgM), anti-NKp30 (F252, IgM), anti-NKp46 (KL247, IgM), anti-KIR3DL1/L2-S1 (AZ158, IgG2a), anti-KIR2DL2/L3 (GL183, IgG1), anti-KIR2DL1/S1 (11PB6 IgG1), anti-NKG2A (Z199, IgG2b), anti-p75 (QA79, IgG1), anti-IRp60 (E59/126, IgG1), anti-LFA-1 (ECM17/120, IgM), anti-LFA-3 (TS2/9, IgG1), anti-CD16 (c127, IgG1), anti-HLA-DR (D1.12, IgG2A), anti-PVR (M5A10, IgG1), anti-Nectin-2 (L14, IgG2a), anti-MIC-A (BAM195, IgG1), anti-ICAM-1 (7E22, IgG1), anti-CD69 (c227, IgG1), anti-CD25 (MAR93, IgG1), anti-NKp44 (Z231, IgG1), anti-CD86 (FM95, IgG1), anti-CD1a (FM184, IgM).

The following commercial mAbs were also used: anti-CD62L (clone DREG-56, IgG1) mAb, anti-CCR3 (clone 61828, IgG2A) mAb, PE-conjugated IgG2A-specific goat anti-rat secondary reagents (BD Biosciences, San Jose, CA, USA); anti-CXCR1 (IgG1) (Santa Cruz, CA, USA); anti-CCR4 (IgG1) (BD Pharmingen); anti-CXCR4 (IgG2b) (R&D); anti-ICAM2 (clone B-T1), anti-ICAM3 (clone BR1) (Diaclone); anti-CD32 (IgG2a) (Beckman Coulter); anti-ULBP1 (clone M295), anti-ULBP2 (clone M310) and anti-ULBP3 (clone M550) (Amgen Inc., Seattle, WA, USA). Anti-PD-L1 and anti-PD-L2 (IgG1) were kindly provided by Prof. Daniel Olive (Aix Marseille Université, France).

Annexin V-FITC was purchased from Bender MedSystems (Vienna, Austria, Europe). ToPro3 Iodide was purchased from Invitrogen (Eugene, OR, USA). Cytofluorimetric analysis of eosinophlis was performed by gating on Annexin V^−^/ToPro3^−^ cells. Anti-B7-H6 (IgG1) was kindly provided by Prof. Eric Vivier (Centre d’Immunologie de Marseille-Luminy, France). Anti-human IFNγ was purchased from R&D Systems Inc. (Minneapolis, MN, USA). Cytofluorimetric analysis was assessed by flow cytometry FACSCalibur; Becton Dickinson & Co. (Mountain View, CA, USA).

### Isolation and Culture of Human Leukocytes

Buffy coats from healthy donors were obtained from the Immunohematology and Transfusion Center at the S. Martino Hospital (Genova, Italy).

Approval was obtained by the ethical committee of IRCCS S. Martino-IST (39/2012) of Genova (Italy). Informed consent was provided according to the Declaration of Helsinki.

Buffy coats were mixed at ratio 1:1 with 2% Dextran T500 (Pharmacosmos, Holbaek, Denmark). After sedimentation of red blood cells, the upper phase was separated into granulocytes and mononuclear cells by density gradient centrifugation. Residual erythrocytes in the pellet were gently lysed in water to yield a pure population of granulocytes. To obtain a pure population of eosinophils from granulocytes, we used the eosinophil isolation Kit (Miltenyi Biotec, Bergisch Gladbach, Germany) according to the manufacturer’s instruction. The purity of eosinophils was greater than 98% (defined as CD16^−^/2B4^+^/NTBA^+^ granulocytes, as shown in Figure S1 in Supplementary Material).

Notably, healthy donors were selected based on the percentage of eosinophils in peripheral blood and on their phenotype after separation. In particular, we discarded donors with a percentage of eosinophils more than 4% and with a phenotype indicating, according to the information found in the literature, a possible activation or sensitization of eosinophils (e.g., expression of CD69).

Purified eosinophils were resuspended in RPMI 1640 medium supplemented with 2 mM glutamine, 50 µg/ml penicillin, 50 µg/ml streptomycin, and 10% heat-inactivated FCS (Sigma-Aldrich, Taufkirchen, Germany), in the absence or in the presence of cytokines (IFNγ 500 U/ml, TNFα 200 U/ml, IL12 1 ng/ml, IL15 20 ng/ml, GM-CSF 50 ng/ml, or IL5 50 ng/ml, all purchased from Peprotech Inc., London, UK) or in the presence of NK cells. Importantly, in all cytofluorimetric analyses, we only considered live eosinophils (Annexin V^−^ and ToPro3^−^ cells).

Myeloid DC were generated from monocytes purified using CD14 MicroBeads human Isolation Kit (Miltenyi Biotec) from PBMC of healthy donors. Monocytes were cultured in RPMI 1640 containing 10% FCS, in the presence of IL4 and granulocyte/macrophage colony-stimulating factor (GM-CSF) (Pepro Tech, London, UK) at final concentrations of 20 and 50 ng/ml, respectively. After 6 days of culture, cells were characterized by the CD14^−^CD1a^+^CD83^−^ phenotype corresponding to immature DCs (iDCs). To generate CD83^+^CD86^+^ mature DCs, iDCs were stimulated overnight (o.n.) with LPS (Sigma-Aldrich) at a final concentration of 1 µg/ml.

Pure populations of NK cells were obtained from PBMC or lymphocytes using the NK cell isolation kit (Miltenyi Biotec, Bergisch Gladbach, Germany) according to the manufacturer’s instruction. In some experiments, MACS CD15 micro beads were added to further improve depletion of granulocytes. The purity of NK cells was greater than 98% NK cells (defined as CD56^+^/CD3^−^).

Such freshly purified NK cells were resuspended in RPMI 1640 medium, supplemented with 2 mM glutamine, 50 µg/ml penicillin, 50 µg/ml streptomycin, and 10% heat-inactivated FCS (Sigma-Aldrich, Taufkirchen, Germany), in the presence of either 1 ng/ml of IL12 (purchased from Peprotech Inc., London, UK) or 20 ng/ml of IL15 (purchased from Peprotech Inc., London, UK). These pro-inflammatory cytokines were selected for their known activating properties on NK cells, and because, unlike IFNγ and GM-CSF, they do not affect eosinophil survival nor eosinophil phenotype (Figure S2 in Supplementary Material) (38–40).

Cells were plated at 10^5^ cells/ml in round-bottom 96-well tissue culture plates (Costar, Corning Corp.). After overnight culture (o.n.), NK cells were washed and incubated o.n. with purified eosinophils. Then, NK cells were harvested and assessed for surface phenotype, cytolytic activity and cytokine production. For cytofluorimetric analyses, NK cells and eosinophils were first identified on the basis of their size difference (FSC) and granularity (SSC) and then of different surface markers. In particular, NK cells were identified as CD56^+^/CD3^−^ cells, whereas eosinophils were identified as CD16^−^/2B4^+^ granulocytes. Dead cells were defined as Annexin V^+^/ToPro3^+^ cells, thus, cytofluorimetric analysis of eosinophlis was always performed by gating on Annexin V^−^/ToPro3^−^ cells.

In some experiments, the same NK cells, after exposure to eosinophils, were cocultured o.n. with iDCs. Then, DCs were harvested and maturation markers were assessed by flow cytometry. This analysis was performed by gating on CD1a^+^/ToPro3^−^ cells.

For transwell (TW) experiments, NK cells and eosinophils were placed in 24-well TW (0.3 µm pore size; Corning Costar), upper and bottom chamber, respectively. To obtain activated polyclonally expanded NK cells (bulk), freshly isolated NK cells were cultured on irradiated feeder cells in the presence of 100 U/mL recombinant human IL2 (Proleukin; Chiron) and 1.5 ng/ml phytohemagglutinin (PHA) (GIBCO Ltd.).

### Production of Soluble Receptors and Immunofluorescence

Plasmids utilized for expression of human NKp30-Fc*, NKp46-Fc*, and DNAM-1-Fc* recombinant molecules were prepared as previously described (41–43), utilizing pRB1-2B4Fcmut vector (kindly provided by M. Falco, Istituto G. Gaslini, Genova, Italy) that contains a mutagenized sequence coding for a human IgG1 portion that does not bind to Fc receptors. Soluble receptors were produced in HEK293T cell line (human embryonic fibroblast) and purified by affinity chromatography using Protein A-Sepharose 4 Fast Flow (Amersham Biosciences) (41–43). Eosinophils (1 × 10^5^ cells) were incubated with 2 µg of NKp30-Fc* and NKp46-Fc* soluble receptors for 30 min at 4°C, washed and stained with PE-conjugated F(ab′)2 goat anti-human IgG (Jackson ImmunoResearch Laboratories) for 30 min at 4°C. Flow cytometry was performed using a FACSCalibur flow cytometer (BD Biosciences) and cells were analyzed with CellQuest Pro software (BD Biosciences).

### RT-PCR Analysis

Total cellular RNA was extracted from eosinophils and from HEK293T cell line (human embryonic fibroblasts) using an RNAeasy Micro Kit (Qiagen, Hilden, Germany). Oligo (dT)-primed cDNA was prepared by standard technique using Transcriptor (Roche, Monza, Italy). Amplification of B7-H6 cDNA (nt. 247-708, Accession N° NR_026750) was performed with Platinum Taq (Life Technologies Paisley, UK) for 35 cycles (30 s at 95°C, 30 s at 58°C, and 1 min at 68°C) using the following primers: H6 for 2 5′ TGCTGTGGGCGCTGACGA and H6 rev2 5′ GGTAGAACCCACTTGACTCA. β-actin and CD63 amplifications were performed for 30 cycles using the same conditions and served as internal controls; primers used were: β-actin up 5′ ACTCCATCATGAAGTGTGACG; β-actin dw 5′ CATACTCCTGCTTGCTGATCC; CD63 up 5′ CAGCCATGGCGGTGGAAG and CD63 dw 5′ CCACTCCCCCAGATGAGG. PCR products were run on a 0.8% agarose gel and visualized by ethidium bromide staining (16, 44).

### Cytolytic Activity

NK cells that had been exposed to IL12 or IL15 and then cocultured with eosinophils were tested for cytolytic activity against various NK cell-susceptible target cells, including K562 and allogeneic iDCs, in a classical 4-h ^51^Cr-release assay as previously described (22). In other experiments, the cytolytic activity of resting or cytokines-primed NK cells was evaluated against autologous or allogeneic freshly isolated eosinophils in a 4-h ^51^Cr-release assay (22). The concentration of mAbs used for masking experiments was 10 µg/ml. The *E*/*T* ratios are indicated in the figure legends.

### Cytokine Production

ELISA kits were used for measuring IFNγ assessment in the supernatants of NK cells stimulated with eosinophils (BioSource Int. Inc., CA, USA). Ab-mediated blocking experiments were performed adding saturating amounts of purified anti-NKp46, anti-NKp30, anti-2B4, and anti-LFA1 mAbs at the onset of the cell cultures.

### Statistical Analysis

Independent samples *t-*test was employed for evaluating quantitative variables. The test is a statistical technique that is used to analyze the mean comparison of two independent groups. The statistical level of significance was preset at 0.05. Graphic representation and statistical analyses were performed using the PASW Statistic version 18.0 software (formerly SPSS Statistics) (IBM, Italy) and GraphPad Prism 6 (GraphPad Software, La Jolla, CA, USA).

## Results

### Eosinophils Express Ligands for NK Cell Receptors

**Figure 1:**
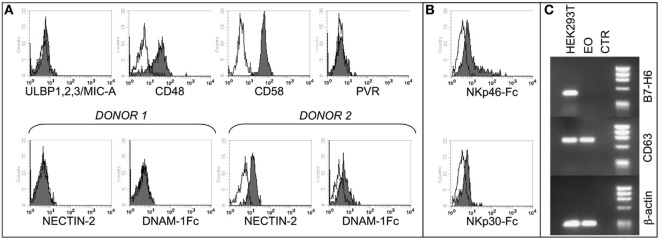
**Analysis of NK cell receptor ligands on eosinophils (EOs) freshly purified from healthy non-atopic donors**. **(A)** EOs were analyzed for the cell surface expression of NK cell receptor ligands including ULBP1,2,3, MIC-A, CD48, CD58, and PVR. A representative donor out of 15 is shown (upper line). The expression of Nectin-2 and, as control, the staining with DNAM-1-Fc* soluble molecule are shown for two representative donors out of 20 analyzed (lower line). Open profiles indicate staining with the corresponding PE-conjugated anti-mouse or anti-human secondary reagents. **(B)** EOs were analyzed for the cell surface binding of NKp30-Fc* and NKp46-Fc* soluble molecules. Open profiles indicate staining with PE-conjugated anti-human IgG secondary reagent. A representative donor out of six is shown. **(C)** B7-H6 mRNA expression was assessed by RT-PCR in EOs and in HEK293T cell line. PCR products were run on a 0.8% agarose gel and visualized by ethidium bromide staining. RT-PCR was also performed with primers specific for CD63 and β-actin as positive controls.

In agreement with previous studies, we found that eosinophils from non-atopic healthy donors express 2B4, NTBA, and IRp60 receptors (45–47), while they do not express CD16 (Figure S1A in Supplementary Material) (48) and CD69 (49). Moreover, eosinophils expressed different chemokine receptors, including CD62L as well as CCR3 (50) and the ligands for the programmed cell death protein 1 (PD-L1 and PD-L2) (Figure S1B in Supplementary Material). To assess the possibility that NK cells and eosinophils may interact with each other, we analyzed the phenotype of resting eosinophils also for the surface expression of specific ligands for NK cell receptors. As shown in Figure 1A, eosinophils did not express ligands for NKG2D (MICA, ULBPs) or for the DNAM-1 receptor (PVR and Nectin-2), although some variations in Nectin-2 expression could be observed among different donors. On the other hand, eosinophils expressed the ligands for the activating coreceptors 2B4 (CD48), CD2 (CD58), and NTBA (NTBA itself) (Figure 1A; Figure S1B in Supplementary Material) (43). Regarding the LFA-1 ligands, freshly isolated eosinophils expressed ICAM-3, whereas they were negative for ICAM-1 and ICAM-2 adhesion molecules (Figure S1B in Supplementary Material).

In order to assess the expression of NCR ligands on eosinophils, we used soluble NKp46-Fc* and NKp30-Fc* molecules. As shown in Figure 1B, both molecules react, albeit weakly, with eosinophils, thus indicating that these cells may express one or more ligands for NKp46 and NKp30.

In order to find out whether the NKp30 ligand expressed on eosinophils is the B7-H6 molecule [whose expression has been recently described on neutrophils/monocytes under inflammatory conditions (51)], we used specific anti-B7-H6 mAbs and performed the analysis at the mRNA level by RT-PCR. These experiments indicate that fresh eosinophils, similar to iDCs, do not express B7-H6 mRNA nor surface B7-H6 protein (Figure 1C and not shown), thus suggesting the existence of alternative (additional) NKp30 ligand/s on these cells. Unfortunately, we could not evaluate the expression of BAG6, due to the unavailability of specific reagents. Collectively, these data demonstrate that eosinophils express several ligands for NK receptors, suggesting that the two innate cell types may interact and influence each other.

### NK Cells Upregulate CD69 Expression and Their Antitumor Cytotoxicity after Direct Contact with Eosinophils

**Figure 2:**
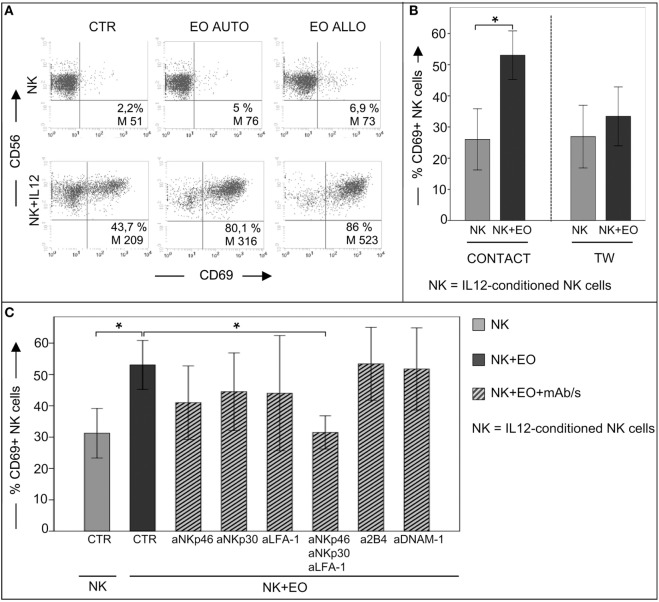
**Analysis of CD69 surface expression on IL12-conditioned NK cells after coculture with eosinophils (EOs) freshly purified from healthy non-atopic donors**. **(A)** Resting NK cells or IL12-conditioned NK cells were cultured o.n. with either autologous (AUTO) or allogeneic (ALLO) EOs and then tested for CD69 expression. The NK/EOs ratio in the coculture was of 1 to 1. Surface expression of CD69 on NK cells was analyzed by gating on CD56^+^/CD3^−^ cells. The percentage and median value of CD69^+^ NK cells are shown for one representative donor out of 10 analyzed. **(B)** IL12-conditioned NK cells were cocultured with allogeneic EOs, either in the presence of cell-to-cell contact (left) or in transwell (right). After o.n. incubation, NK cells were harvested and analyzed for CD69 expression. Gray bars represent CD69 expression on NK cells cultured alone; black bars refer to CD69 expression on NK cells cocultured with EOs. The NK/EOs ratio in the coculture was of 1:1. The average of six independent experiments is shown (% ±SD). *P < 0.05. **(C)** IL12-conditioned NK cells were cocultured with EOs in the absence or in the presence of the indicated blocking mAbs; after o.n., NK cells were harvested and analyzed for CD69 expression. Gray bar represents CD69 expression on NK cells cultured alone, black bar refers to CD69 expression on NK cells cocultured with EOs, ribbed bars represent CD69 expression on NK cells cocultured with EOs in the presence of the indicated blocking mAbs. NK/EOs ratio in the coculture was of 1:1. The average of six independent experiments is shown (% ±SD). *P < 0.05.

We next analyzed whether eosinophils could modulate the NK cell phenotype and effector function. In these experiments, NK cells, either resting or short-term primed with IL12 or IL15, were cocultured o.n. with fresh autologous or allogeneic eosinophils. After o.n. coculture, NK cells were assessed for the expression of the early activation marker CD69. As shown in Figure 2A, the surface density and the percentage of CD69^+^ NK cells were strongly upregulated on IL12-conditioned NK cells, but not on resting NK cells, in the presence of autologous or allogeneic eosinophils. The same results were obtained after stimulation of NK cells with IL15 (not shown). Importantly, CD69 upregulation was mainly detected following cell-to-cell contact, although a slight increase of CD69 expression was detectable also when NK cells and eosinophils were separated by a TW membrane (Figure 2B). In order to identify molecules that may be involved in the interaction between NK cells and eosinophils, cocultures were performed in the presence of mAbs specific for different NK receptors, including the adhesion molecule LFA-1 and the activating receptors NKp46, NKp30, 2B4, and DNAM-1. As shown in Figure 2C, the eosinophil-induced upregulation of CD69 on NK cells was reduced by the combined antibody-dependent blockade of NKp46, NKp30, and LFA-1, but not by masking individual receptors (Figure 2C).

Regarding the expression of other classical activation markers (CD25 and NKp44), no major differences between IL12-conditioned NK cells and IL12-conditioned NK cells in the presence of eosinophils could be detected, although these molecules were weakly increased in NK cells that had been cultured with eosinophils (data not shown).

The level of surface expression of the other molecules analyzed in these experiments (including NKG2D and 2B4) remained substantially similar in NK cells cultured either in the absence or in the presence of eosinophils (data not shown).

**Figure 3:**
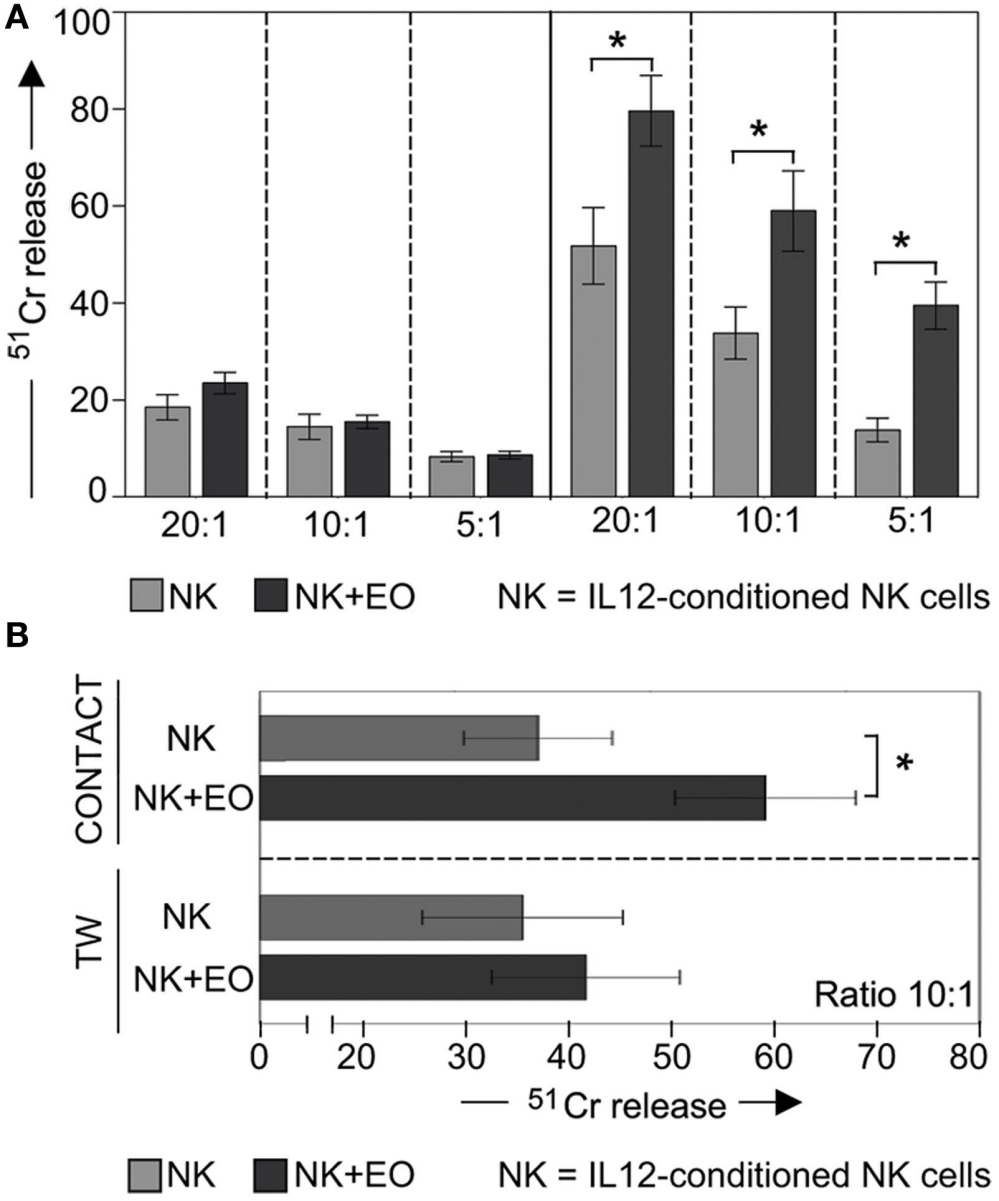
**Anti-tumor cytolytic activity of NK cells cocultured with fresh eosinophils (EOs)**. **(A)** Resting NK cells (left) or IL12-conditioned NK cells (right) were cultured o.n. with EOs at an *E*:*T* ratio of 1:1; then harvested and analyzed for cytolytic activity against K562 at various *E*:*T* ratios. Gray bars refer to lysis by NK cells cultured alone, black bars represent lysis by NK cells cocultured with EOs. The average of six independent experiments is shown (% ±SD). *P < 0.05. **(B)** IL12-conditioned NK cells were cocultured with EOs, either in the presence of cell-to-cell contact (top) or in transwell (bottom). After o.n. culture, NK cells were harvested and analyzed for cytolytic activity against K562 at *E*:*T* ratio of 10:1. Gray bars refer to lysis by NK cells cultured alone, black bars represent lysis by NK cells cocultured with EOs. The average of six independent experiments is shown (% ±SD). *P < 0.05.

In the same set of experiments, NK cells were used as effector cells in cytolytic assays against K562 (a classical NK-susceptible tumor target). As shown in Figure 3A, IL12-stimulated NK cells displayed significantly increase of cytotoxicity after coculture with eosinophils (right). In contrast, under the same culture conditions, resting NK cells did not acquire a higher cytolytic activity against the same target cells (left).

Notably, the stimulatory effect on NK cell cytotoxicity was dependent on cell-to-cell contact, as no increase in cytotoxic activity was observed in TW experiments (Figure 3B).

### NK Cells Release High Amounts of IFNγ after Direct Contact with Eosinophils

**Figure 4:**
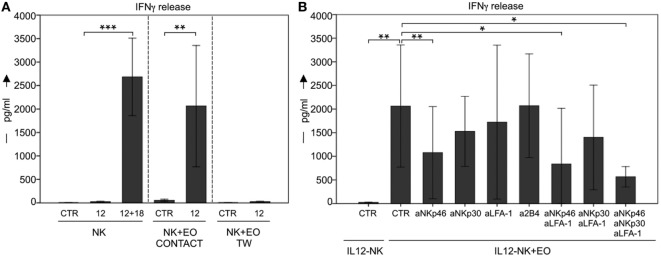
**Analysis of IFN-γ secretion by NK cells after coculture with eosinophils (EOs)**. **(A)** Resting NK cells (CTR) or IL12-conditioned NK cells were cultured o.n. with EOs, either in the presence of cell-to-cell contact or in transwell. After o.n. culture, supernatants were harvested and then analyzed by ELISA for the presence of IFNγ. NK cells that had been exposed o.n. to IL12 plus IL18 were used for comparison. The NK/EOs ratio in the coculture was of 1:1. Average of six independent experiments is shown (pg/ml ± SD). **P < 0.01; ***P < 0.001. **(B)** IL12-conditioned NK cells were cocultured with EOs in the absence or in the presence of the indicated blocking mAbs; after o.n. culture, supernatants were harvested and then analyzed by ELISA for the presence of IFNγ. The NK/EOs ratio in the coculture was of 1:1. Average of six independent experiments is shown (pg/ml ± SD). *P < 0.05; **P < 0.01.

In order to determine whether eosinophils could also promote the production of pro-inflammatory cytokines by NK cells, coculture supernatants were evaluated for the presence of IFNγ (Figure 4). In these experiments, eosinophils did not induce IFNγ production by resting NK cells. However, high amounts of this cytokine was detected in NK cells pre-activated o.n. with IL12. In line with the results above, there was no detectable IFNγ production by NK cells in the absence of NK/eosinophil direct contact (Figure 4A). In all these experiments, resting NK cells that had been exposed o.n. to IL12 plus IL18 were used as positive control.

In experiments aimed at defining the molecular interactions involved in the eosinophil-NK cells cross talk, cocultures were performed in the presence of mAbs specific for different NK receptors. As shown in Figure 4B, antibody-mediated masking of NKp46 and even more when used in combination with anti-LFA-1 mAb inhibited IFNγ release by NK cells. Remarkably, the maximal effect of inhibition occurred again upon simultaneously masking of NKp46, LFA-1, and NKp30 (Figure 4B). By contrast, masking of other receptors (e.g., 2B4) had no substantial effect (Figure 4B and not shown).

Thus, both TW and masking experiments pointed to a critical role for receptor/ligand interactions during the NK/eosinophil cross talk, resulting in amplification of NK cell activation, as determined by the upregulation of CD69 expression and by the increases of cytotoxic activity and IFNγ production.

### NK Cells Exposed to Eosinophils Acquire a Higher Capacity to Kill Myeloid iDCs and to Induce Their Maturation

**Figure 5:**
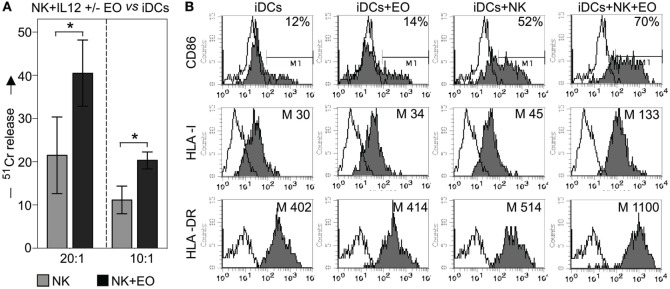
**NK cells exposed to eosinophils (EOs) display a higher capability to kill myeloid immature DCs (iDCs) and to induce their maturation**. **(A)** IL12-conditioned NK cells were cultured o.n. with EOs at an *E*:*T* ratio of 1:1, then harvested and analyzed for cytolytic activity against iDCs at various *E*/*T* ratios. Gray bars refer to lysis by NK cells cultured alone, black bars represent lysis by NK cells cocultured with EOs. The average of six independent experiments is shown (% ±SD). *P < 0.05. **(B)** IL12-conditioned NK cells were cultured o.n. with EOs at an *E*:*T* ratio of 1:1; the same NK cells were then cocultured with iDCs. After o.n., dendritic cells (DCs) were harvested and assessed by flow cytometric analysis for maturation markers such as CD86, HLA-I, and HLA-DR. For comparison, iDCs were cultured either alone or with EOs and NK cells separately. These analyzes were performed by gating on CD1a^+^/ToPro3^−^ cells. Open profiles indicate staining with PE-conjugated anti-mouse IgG secondary reagent. The percentage of CD86 and the median value of HLA-I and HLA-DR on DCs are shown for a representative donor out of six analyzed.

Next, we investigated whether coculture with eosinophils could promote NK cell-mediated killing of iDCs. In agreement with previous data, exogenous IL12-conditioned NK cells were able to kill iDCs (22); however, this activity was significantly increased after coculture with eosinophils, as shown in Figure 5A.

Next, we evaluated whether the interaction with eosinophils could influence the NK cell capability of promoting DC maturation. To this end, IL12-conditioned NK cells and eosinophils were cocultured o.n., then harvested, washed, and cultured with iDCs. After 24 h, the expression of CD86, HLA class-I and HLA class-II (i.e., HLA-DR) molecules on DCs was determined by cytofluorimetric analysis. As shown in Figure 5B, substantial increments in the mean expression of HLA molecules (both class I and II) and in the percentage of CD86-expressing cells were detected when iDCs were cocultured with NK cells plus eosinophils, as compared to iDCs cocultured with NK or eosinophils alone. In some experiments, eosinophils were removed before culturing NK cells with iDCs and also under these conditions DCs could undergo maturation (not shown).

### NK Cells Activate Eosinophils to Acquire Both Migratory Potential and the Phenotypic Features of APCs

**Figure 6:**
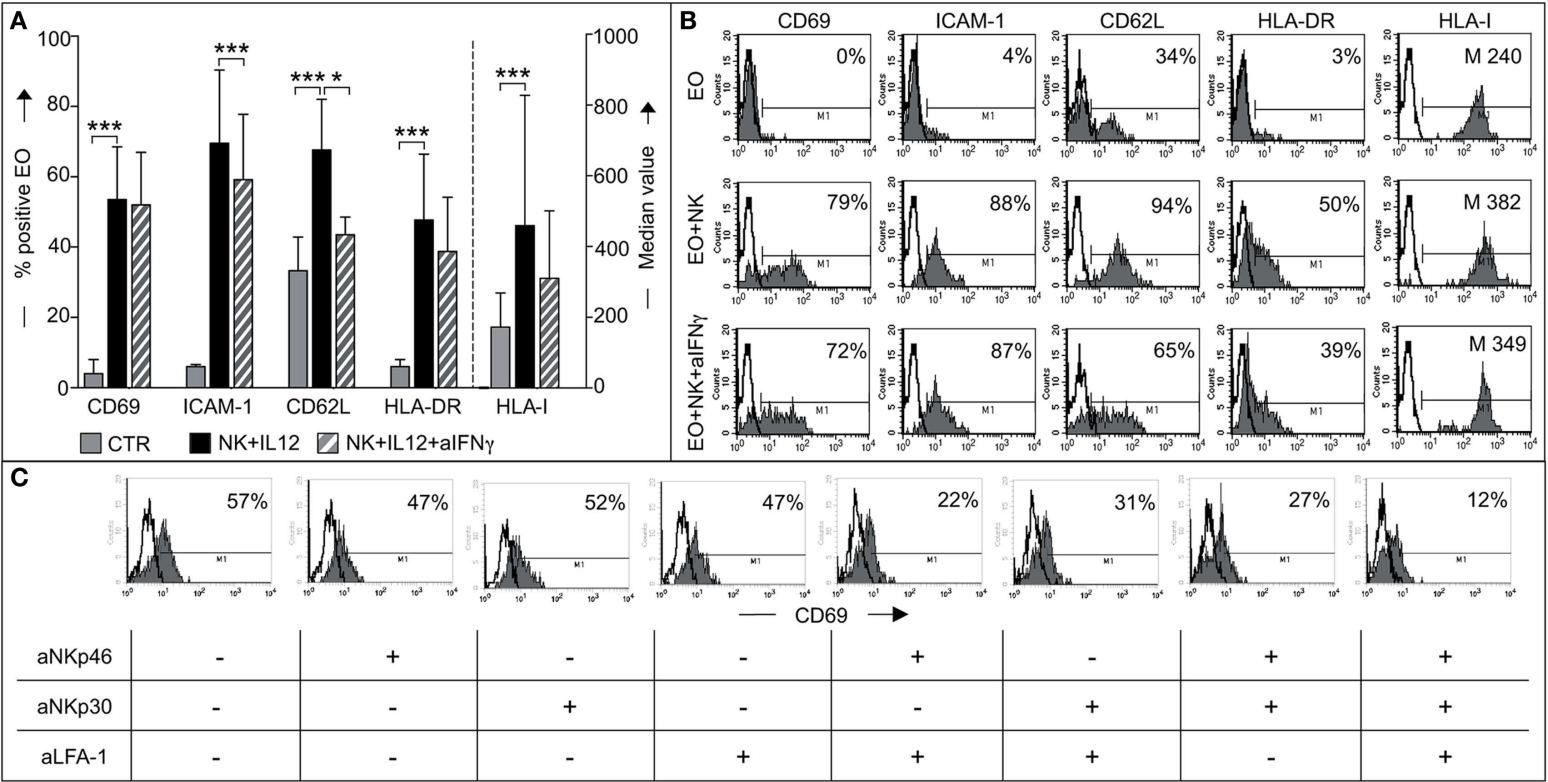
**Analysis of eosinophil (EO) activation induced by IL12-conditioned NK cells**. **(A)** EOs were cultured alone (CTR) or with IL12-conditioned NK cells at an *E*:*T* ratio of 1:1 in the presence or in the absence of neutralizing anti-IFNγ mAbs. After o.n. culture, EOs were harvested and assessed by flow cytometric analysis for maturation markers such as CD69, ICAM-1, CD62L, HLA-DR, and HLA-I. Surface expression of these markers on EOs was analyzed by gating on Annexin V^−^/ToPro3^−^ cells. The percentage of viable EOs (Annexin V^−^/ToPro3^−^) was 30–55% in CTR, 70–85% in cocultures containing NK + IL12 and 60–71% in those containing NK + IL12 + anti-IFNγ. The bars indicate the percentage of positive cells and the median value of HLA-I+ EOs. Gray bars refer to EOs cultured alone, black bars represent EOs cocultured with NK + IL12 cells and striped bars refer to EOs cocultured with NK + IL12 cells in the presence of neutralizing anti-IFNγ mAb. The average of six independent experiments is shown (% ±SD). *P < 0.05; ***P < 0.001. **(B)** Surface expression of the markers analyzed in panel **(A)** is shown for one representative donor out of 6 analyzed. Open profile indicates staining with PE-conjugated anti-mouse IgG secondary reagent. The percentage of CD69, ICAM-1, CD62L, HLA-DR, and the median value of HLA-I are indicated. **(C)** EOs cells were cultured with IL12-conditioned NK cells at an *E*:*T* ratio of 1:1 in the presence (+) or in the absence (−) of the indicated blocking mAbs; after o.n. culture, EOs were harvested and assessed by flow cytometric analysis for CD69 expression by gating on AnnexinV^−^/ToPro3^−^ cells. Open profiles indicate staining with PE-conjugated anti-mouse IgG secondary reagent. The percentage of CD69 is shown for one representative donor out of six analyzed.

Next, we investigated whether phenotypic changes in eosinophils occurred following interaction with NK cells. To this end, fresh eosinophils were cocultured with NK cells (either resting or conditioned with IL12/IL15). At the end of the culture period, eosinophils were harvested and analyzed for the expression of a number of informative markers, including CD69, ICAM-1, HLA molecules, and CD62L. A significant *de novo* surface expression of CD69, ICAM-1, and HLA-DR molecules and a marked upregulation of HLA class-I and CD62L molecules was detected on eosinophils cocultured with IL12-conditioned NK cells (Figures 6A,B). The same results were obtained by pre-treating NK cells with IL15. Resting NK cells did not induce any substantial effect (not shown).

Experiments were also performed in the presence of blocking anti-IFNγ mAbs to understand whether the *de novo* expression/upregulation of the above surface molecules could be induced by IFNγ. As shown in Figures 6A,B, when cells were cultured in the presence of anti-IFNγ mAbs, the expression of most of the above markers was reduced but not abolished (Figure 6B).

This indicates that the modification of eosinophil phenotype induced by cytokine-treated NK cells is in part, but not exclusively, due to the production of IFNγ during coculture. Next, we determined if the same receptor/ligand interactions responsible for eosinophil-mediated induction of NK cell effector functions were also involved in the events leading to eosinophil activation. To this end, neutralizing mAbs specific for NKp46, NKp30, or LFA-1 were added, alone or in combination, to cocultures and the expression of CD69 on eosinophils analyzed. As shown in Figure 6C, mAbs, added individually, did not (or only modestly) inhibit CD69 expression. In contrast, CD69 expression was significantly decreased in eosinophils when the neutralizing mAbs specific for NK receptors were used in combination. Particularly strong inhibition was obtained when anti-NKp46, anti-NKp30, and anti-LFA-1 mAbs were simultaneously added to the coculture.

### NK Cells Are Capable of Killing Both Autologous and Allogeneic Eosinophils *via* NKp46 and NKp30

**Figure 7:**
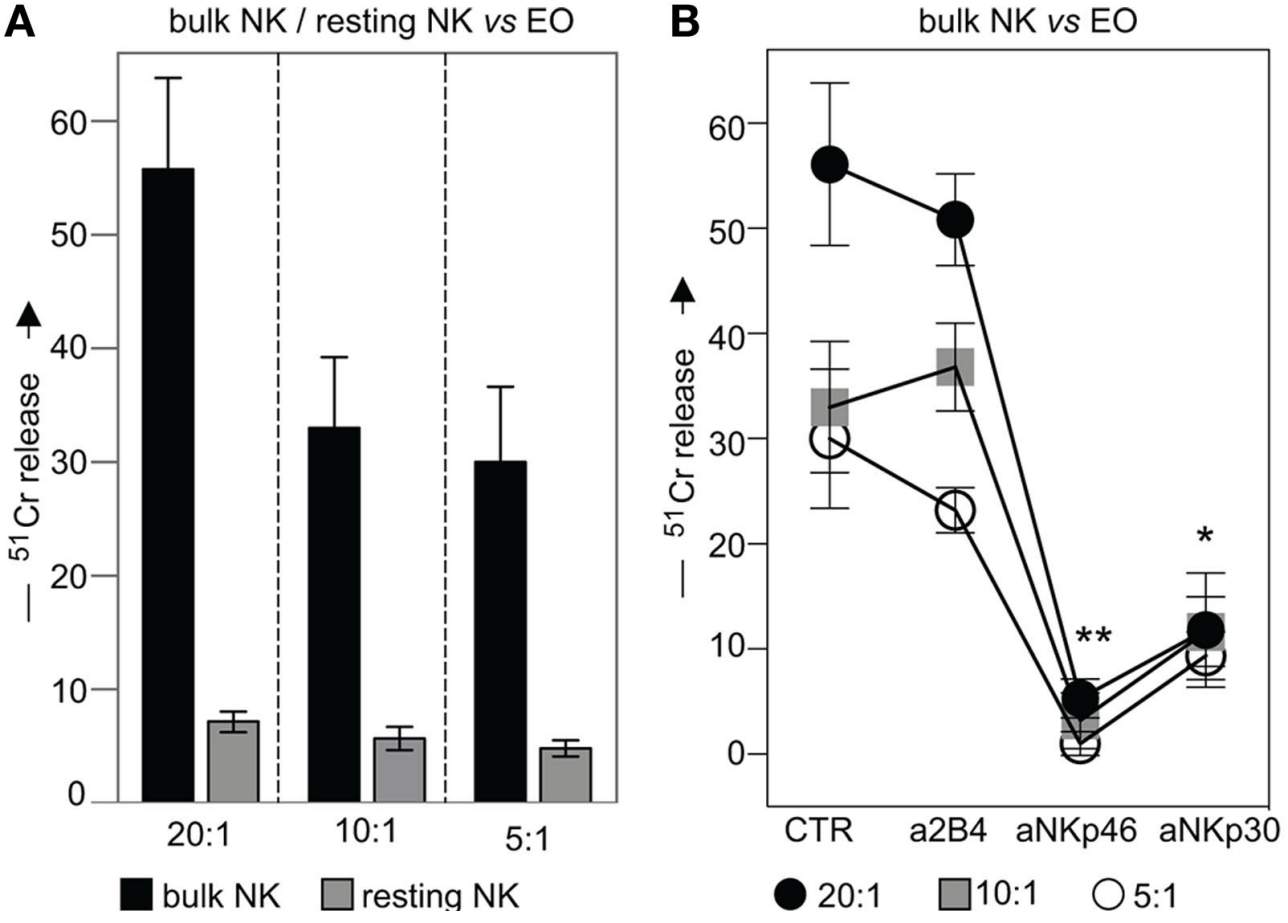
**Cytolytic activity of IL2-conditioned or resting NK cells against eosinophils (EOs) freshly purified from healthy non-atopic donors**. **(A)** IL2-conditioned (bulk) or resting NK cells were tested in ^51^Cr-release assay against EOs at various *E*:*T* ratios. Black bars represent lysis by bulk NK cells; gray bars refer to lysis by resting NK cells. The average of six independent experiments is shown (% ±SD). **(B)** IL2-conditioned NK cells (bulk) were analyzed for their cytolytic activity against EOs in the absence or in the presence of the indicated blocking mAbs at various *E*:*T* ratios. Black circles: *E*:*T* ratio of 20:1; gray squares: *E*:*T* ratio of 10:1; white circles: *E*:*T* ratio of 5:1. The average of six independent experiments is shown (% ±SD). *P < 0.05; **P < 0.01.

To investigate whether eosinophils could represent possible targets for NK cell cytotoxicity, fresh (not-activated) allogeneic eosinophils were exposed either to resting or to IL2-activated NK cells (bulk) in ^51^Cr-release cytolytic experiments. As shown in Figure 7A, IL2-activated, but not resting NK cells, displayed a strong cytotoxic activity toward allogeneic eosinophils. In order to evaluate the contribution of one or another activating NK receptor, cytolytic assays were performed in the presence of mAbs specific for major activating NK receptors. As shown in Figure 7B, NKp46 and NKp30 mainly contributed to the killing of eosinophils, since mAb-mediated masking of these receptors, resulted in significant inhibition of lysis, while mAbs directed to other activating NK receptors had no substantial effect.

**Figure 8:**
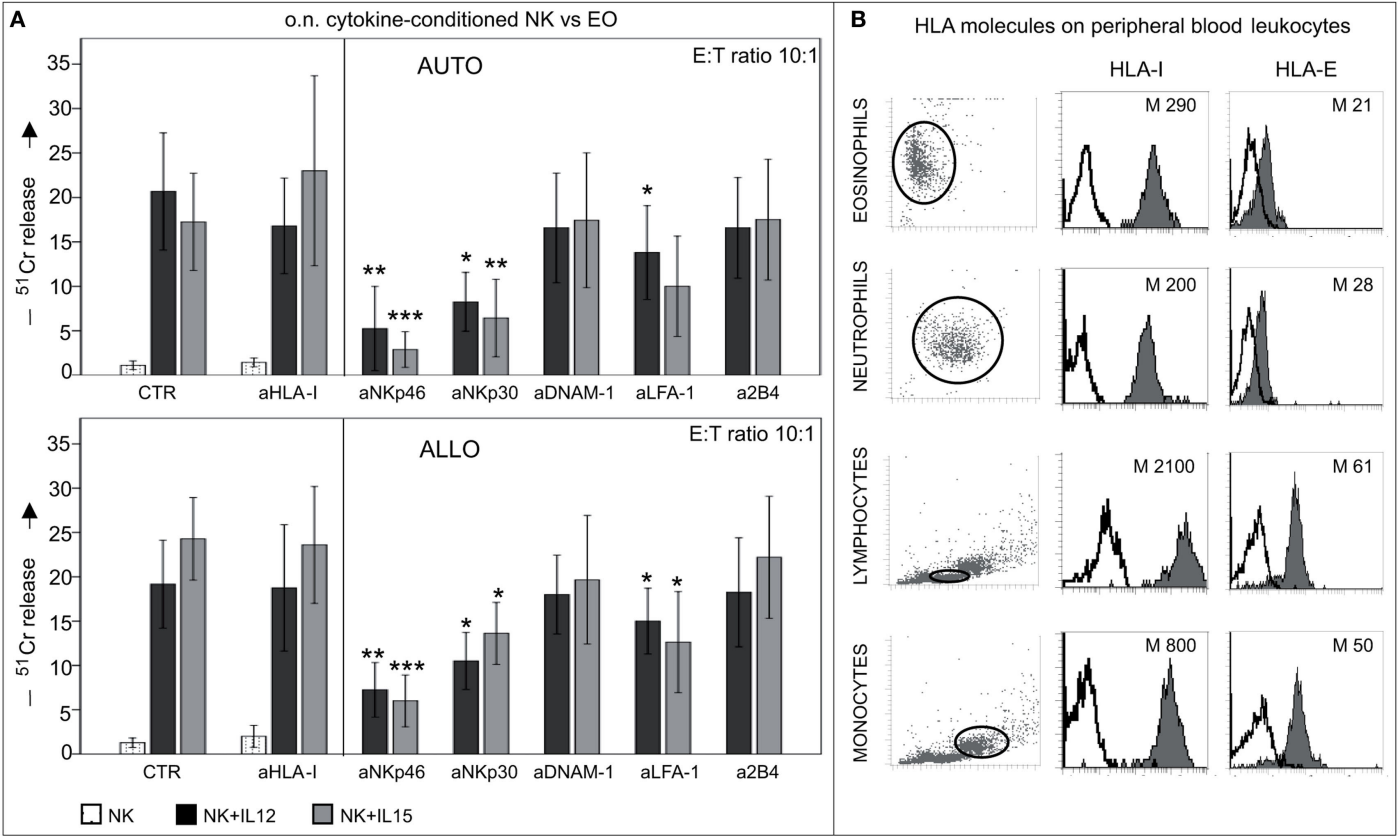
**Cytolytic activity of short-term-conditioned NK cells against autologous (AUTO) or allogeneic (ALLO) eosinophils (EO)**. **(A)** NK cells were cultured o.n. with or without IL12 or IL15 and then tested in ^51^Cr-release assay against autologous (upper panel) or allogeneic (lower panel) EOs. The cytolytic activity was tested in the absence or in the presence of the indicated blocking mAbs at an *E*:*T* ratio of 10:1. White bars refer to lysis by resting NK cells, black bars represent lysis by IL12-conditioned NK cells, gray bars refer to lysis by IL15-conditioned NK cells. The average of eight independent experiments is shown (% ±SD). *P < 0.05; **P < 0.01; ***P < 0.001. *P* value was obtained by comparing the conditions NK + IL12 (black bars) or NK + IL15 (gray bars) in the presence of the different mAbs with the same conditions in the absence of mAbs (CTR). **(B)** Different leukocyte populations derived from peripheral blood of healthy donors were analyzed for surface expression of HLA-I and HLA-E molecules (median values are indicated). A representative donor out of 15 is shown.

Similar results were obtained in cytolytic assays performed in an autologous setting using IL12- or IL15- short-term-primed NK cells as effector cells. As shown in Figure 8A, both types of cytokine-activated NK cells displayed similar levels of cytotoxicity against autologous or allogeneic eosinophils, while NK cells pre-cultured with IL4 or IL18 did not display any cytotoxicity against eosinophils (data not shown) (22). Notably, mAb-mediated disruption of inhibitory receptors/HLA class-I interactions did not result in increases of cytotoxicity (Figure 8A, left). These data suggest that HLA class-I molecules do not provide substantial protection to eosinophils from NK cell-mediated cytotoxicity. In agreement with these results, eosinophils displayed a low expression of surface HLA class-I molecules as compared to other innate cells known to interact with NK cells (Figure 8B).

## Discussion

In the present study, we have analyzed the cross talk occurring between human NK cells and eosinophils. We show that, after direct contact with eosinophils, cytokine-primed NK cells become significantly activated, acquiring the capability of releasing high amounts of IFNγ, killing tumor cells more efficiently, and promoting adaptive immune responses, by killing unfit iDC and favoring the selection of appropriate mDCs. All of these functional activities appear to be primarily consequent to the interaction between NCRs, expressed on NK cells, and surface ligands on eosinophils cell surface. In turn, primed NK cells could strongly influence eosinophils by inducing an APC-like phenotype. In addition, we show that, at high NK/eosinophil ratios, NK cells can efficiently kill both autologous and allogeneic eosinophils, suggesting the existence of NK cell-mediated mechanisms capable of exerting a regulatory control on eosinophil activity.

At the site of infection, activation of immune cells results in the secretion of pro-inflammatory cytokines and chemokines, resulting in the recruitment of different immune cells. Recruited NK cells receive activating signals inducing their effector functions and participate to the functional interactions with other immune cells (15, 17, 21, 22, 52).

Previous studies suggested an important role for the interaction between NK cells and monocyte-derived DCs in both the initiation of the immune response and induction of down-stream adaptive T cell immunity (13, 17, 52–55). For example, activated NK cells acquire the capability of killing iDCs (*via* the NKp30 activating receptor), which do not express adequate amounts of HLA molecules (55). By this mechanism, referred to as “NK cell-mediated editing of DCs,” NK cells may ensure the quality of DCs undergoing maturation. In addition, through the production of soluble factors (such as IFNγ and TNFα) released upon activation, NK cells favor the progression of DC maturation (56). Thus, the final outcome of the “DC editing process” would be the selection of the “fittest” DCs, thanks to the removal of those that, due to the low expression of HLA molecules, would fail to mediate efficient antigen presentation and T-cell priming (18, 22, 57).

As previously shown, additional cell types, including macrophages and neutrophils, that are either resident in tissues or recruited to inflammatory sites, may interact each other and generate a cross talk with NK cells during the early phases of innate immune responses (16, 21, 22, 41, 58, 59). Thanks to the demonstration that eosinophils interact functionally with NK cells, the present study extends the number of innate cells participating in cross talks among cells of the innate immunity. We show that after direct interaction with eosinophils, NK cells undergo activation, release IFNγ, and upregulate the cytotoxic activity against different targets. The eosinophil-induced phenotypic and functional effects on NK cells were to a large extent dependent on close cell-to-cell interaction involving activating NK receptors, including NKp30 and NKp46. Moreover, in agreement with studies on the cross talk between NK and other innate cells, we show that eosinophils can also improve the ability of NK cells to induce DC editing and maturation.

Only few studies have addressed the interaction between NK cells and eosinophils; for example, it has been reported that NK cells may exhibit a chemoattraction toward the eosinophil-released IL8 and that this effect is increased by IL15 (60). Moreover, in allergic rhinitis, NK cells were shown to infiltrate the epithelial layers and the stroma of nasal tissue in response to CX3CL1 and CCL26. In asthmatic patients, a positive correlation was documented between the eosinophil and NK cell numbers and the status of cell activation (61). In addition, two recent studies have suggested that NK cells may promote apoptosis of eosinophils (49, 62), but the molecular mechanisms underlying these events were not addressed. Our study is shedding light on some of these molecular mechanisms and provides evidence that the NK cell cytotoxicity against eosinophils is dependent on NKp46, NKp30, and LFA-1 engagement (Figure 8). In accordance with these data, we found that eosinophils are capable of binding soluble forms of the NKp30 and NKp46 receptors. Interestingly, the NKp30-Fc* binding did not reflect the expression of the recently identified NKp30-ligand, B7-H6, suggesting that eosinophils similar to monocyte-derived DCs (5) express a different cell surface ligand for NKp30.

After coculture with NK cells, eosinophils *de novo* expressed CD69 (an activation marker), and ICAM-1 (important for cellular adhesion), and upregulated CD62L (a receptor involved in the recruitment of eosinophils to the SLCs) and HLA class-I and -II molecules (that confer the capability to present antigens). These events were mainly dependent on cell-to-cell contact, although also IFNγ released by NK cells substantially contributed to this effect, as demonstrated by the partial inhibition detectable in the presence of blocking anti-IFNγ mAb (63). Regarding the expression of CD69, our data are in line with those recently reported by Awad et al. (49), although the culture conditions used by these authors were different. In this context, it is possible that these conditions [i.e., coculture of cells in a Th2 environment (IL-5)] may be responsible for some additional differences in the outcome of the cross talk between NK and eosinophils, including the role of activating NK receptors in the process of recognition and killing of eosinophils.

In the past, eosinophils have been merely considered end-stage cells involved in host protection against parasite infection (64); however, recent studies have changed this perspective and eosinophils are now considered multifunctional leukocytes involved in tissues homeostasis, in innate immune responses to certain pathogens, and in modulation of adaptive immune responses (65–68). In addition, several lines of evidence suggest that eosinophils are capable of producing immunoregulatory cytokines and are actively involved in modulation of T cell responses (66). Remarkably, a role for eosinophils as APCs has recently been proposed (69). In this context, traditionally, eosinophils have been associated with Th2-responses (66, 70–72), in line with their ability to function as APCs and to release Th2-cytokines (28). However, it is important to underline that the majority of these results were obtained with eosinophils derived from atopic or cancer patients. Actually, it is now well established that eosinophils respond to Th1-cytokines, such as IFNγ (63, 73, 74), and their active role in Th1-responses has been proposed (75, 76). In line with this concept, our data suggest that eosinophils derived from healthy donors are capable of driving activated NK cells toward an inflammatory response leading to an effective “editing” of DCs, resulting in induction of Th1 (and not Th2) responses. Our data also suggest that NK cells can activate eosinophils to express or upregulate CD69, ICAM-1, CD62L, and HLA molecules, which may favor their migration toward SLCs, where they can present antigens to T cells (66).

**Figure 9:**
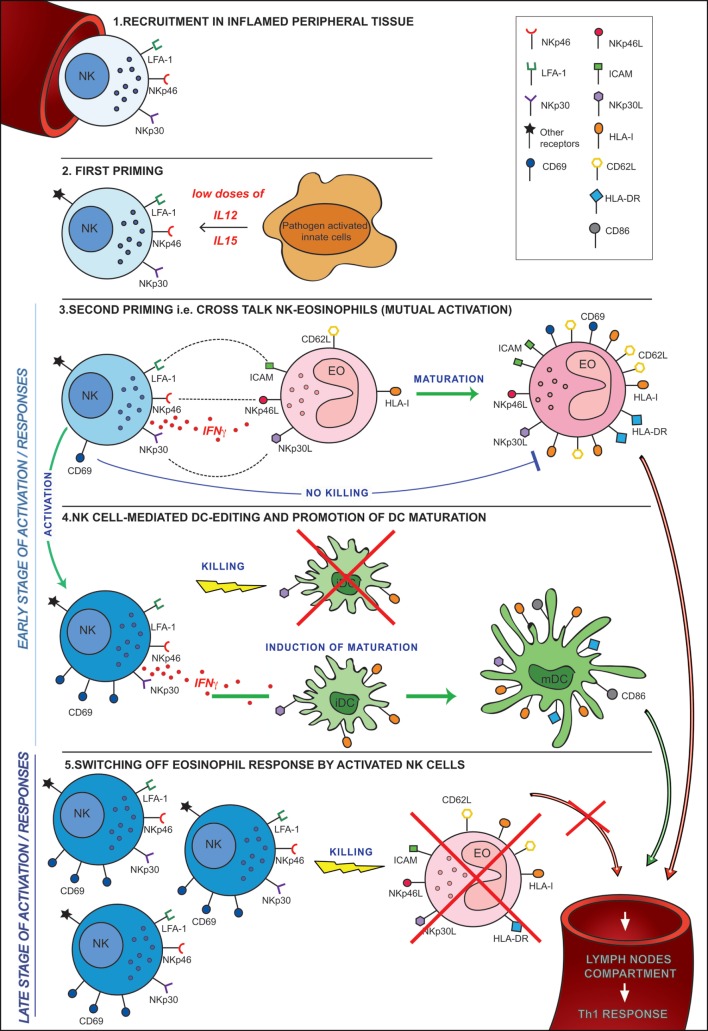
**Hypothesis of cross talk between NK cells and eosinophils (EOs) in an inflammatory microenvironment**. 1. Recruitment in peripheral tissues. 2. First priming. This can occur following the release of pro-inflammatory cytokines (i.e., IL12 and IL15) by resident innate cells activated by pathogens in the inflammatory microenvironment. 3. Second priming, i.e., cross talk NK-EO (mutual activation). Full priming takes place when NK cells interact with EOs. During this cross talk, NK cells achieve optimal activation in terms of CD69 expression, cytokine release, and cytotoxicity. At the same time, EOs acquired an activated phenotype becoming able to migrate into lymph nodes where they may strengthen/induce a Th1 response. 4. NK cell-mediated dendritic cell (DC) editing and promotion of DC maturation. The EO-mediated NK cell activation allows the promotion of the mechanisms involved in DC editing and maturation. These events are crucial for the selection of the “most fitting” DCs for antigen presentation and T-cell priming. 5. Switching off EO response by activated NK cells. During a late stage of activation/response, activated NK cells, that are now outnumbering EOs, kill non-activated EOs, avoiding their migration into lymph nodes thus preventing unwanted antigen presentation.

In conclusion, our study provides novel information on the molecular mechanisms involved in the cross talk between eosinophils and NK cells, demonstrating that these interactions are mediated mainly by NCR/NCR ligand interactions. Our results also show that, upon engagement of these receptors, NK cells that had been exposed to innate cytokines, amplify their effector function against tumor cells and DCs. These innate cytokines are primarily released by other players of innate immune responses recruited at the same inflammatory sites. In addition, NK cells upon encountering eosinophils may either release IFNγ and promote their maturation toward an APC-like migratory cell or, on the contrary, may kill them terminating their activity and contributing to dampening an excessive inflammatory response (Figure 9).

## Ethics Statement

Buffy coats from healthy donors were obtained from the Immunohematology and Transfusion Center at the S. Martino Hospital (Genova, Italy). Approval was obtained by the ethical committee of IRCCS S. Martino-IST (39/2012) of Genova (Italy). Informed consent was provided according to the Declaration of Helsinki.

## Author Contributions

SP designed and performed research and interpreted data; CC and CP did RT-PCR analysis and provided soluble receptors; LM and FT revised the article; AM designed research and interpreted data, EM designed and performed research, interpreted data, and wrote the article.

## Conflict of Interest Statement

AM is a founder and shareholder of Innate-Pharma (Marseille, France). The remaining authors declare no competing financial interests.

## Supplementary Material

Figure S1 **Phenotypic analysis of eosinophils (EOs) freshly separated from healthy non-atopic donors**. **(A)** EOs were purified from peripheral blood of healthy non-atopic donors. The purity of EOs was confirmed by analyzing the phenotype of cells obtained after separation. In particular, EOs expressed omogeneously the phenotype CD16^−^/2B4^+^/NTBA^+^ (left panel), while neutrophils expressed omogeneously the phenotype CD16^+^/2B4^−^/NTBA^−^ (right panel). Dead cells were defined as Annexin V^+^/ToPro3^+^ cells, thus cytofluorimetric analysis of EOs was always performed by gating on Annexin V^−^/ToPro3^−^ cells. **(B)** EOs were assessed by flow cytometric analysis for a number of surface molecules by gating on AnnexinV^−^/ToPro3^−^ cells. The phenotypic analysis of neutrophils was used for comparison. The data shown in the table were obtained from the analysis of 20 different healthy donors.

Figure S2 **Analysis of survival and activation of eosinophils (EOs) purified from healthy non-atopic donors after culture in the presence of selected cytokines**. **(A)** EOs were cultured in the absence or in the presence of cytokines (IL5, GM-CSF, IFNγ, TNFα, IL12, IL15), then harvested and assessed by flow cytometric analysis for survival markers such as Annexin V and ToPro3. The percentage of Annexin V^−^/ToPro3^−^ EOs is indicated for one representative donor out of 30 analyzed. **(B)** EOs were cultured in the absence or in the presence of cytokines (IL5, GM-CSF, IFNγ, TNFα, IL12, IL15), then harvested and assessed by flow cytometric analysis for the expression of CD69 surface molecules by gating on Annexin V^−^/ToPro3^−^ cells. The bars indicate the percentage of CD69^+^ EOs. The average of 20 independent experiments is shown (% ±SD). **P* < 0.05; ***P* < 0.01. *P* value was obtained by comparing the conditions in the presence of the different cytokines with the condition in the absence of cytokines (CTR).
