# Supplementary figures and images for: The Innate Immune Cross Talk between NK Cells and Eosinophils Is Regulated by the Interaction of Natural Cytotoxicity Receptors with Eosinophil Surface Ligands
Source: Front Immunol. 2017 Apr 28;8:510. doi: 10.3389/fimmu.2017.00510 (PMC5408020; doi:10.3389/fimmu.2017.00510)

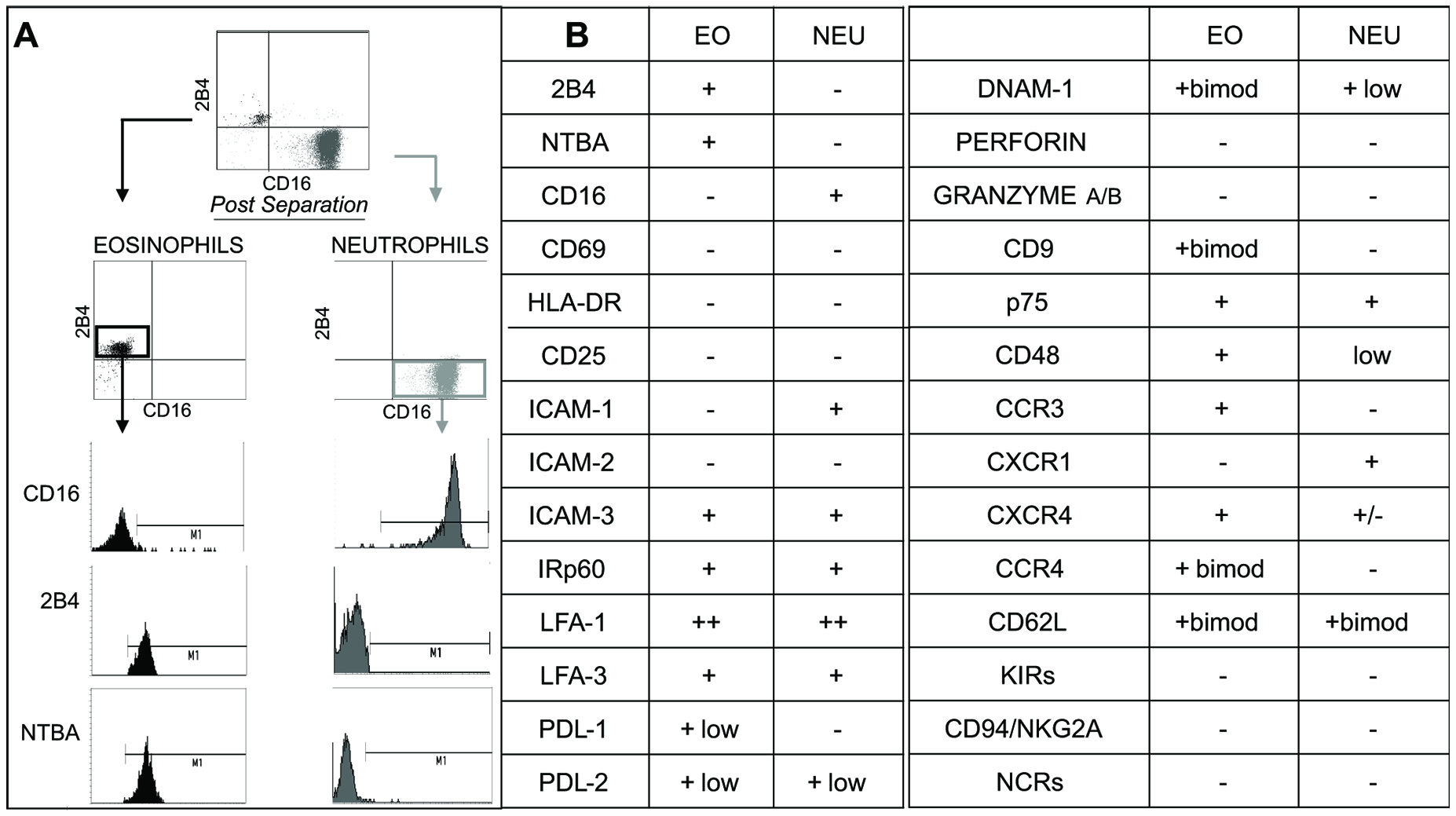

Supplement: Figure S1 — Phenotypic analysis of eosinophils (EOs) freshly separated from healthy non-atopic donors. (A) EOs were purified from peripheral blood of healthy non-atopic donors. The purity of EOs was confirmed by analyzing the phenotype of cells obtained after separation. In particular, EOs expressed omogeneously the phenotype CD16−/2B4+/NTBA+ (left panel), while neutrophils expressed omogeneously the phenotype CD16+/2B4−/NTBA− (right panel). Dead cells were defined as Annexin V+/ToPro3+ cells, thus cytofluorimetric analysis of EOs was always performed by gating on Annexin V−/ToPro3− cells. (B) EOs were assessed by flow cytometric analysis for a number of surface molecules by gating on AnnexinV−/ToPro3− cells. The phenotypic analysis of neutrophils was used for comparison. The data shown in the table were obtained from the analysis of 20 different healthy donors. [file image_1.tif]

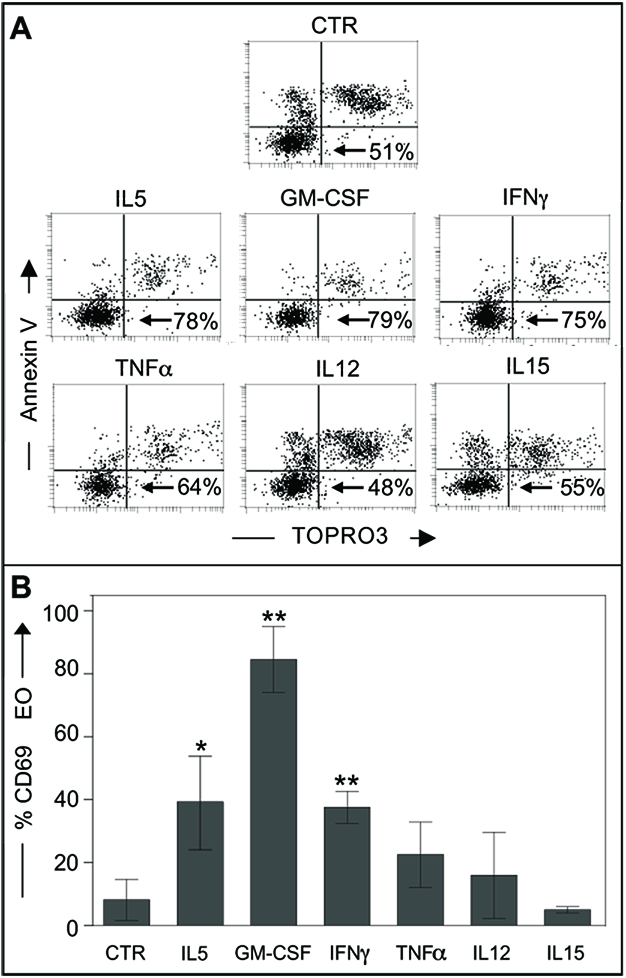

Supplement: Figure S2 — Analysis of survival and activation of eosinophils (EOs) purified from healthy non-atopic donors after culture in the presence of selected cytokines. (A) EOs were cultured in the absence or in the presence of cytokines (IL5, GM-CSF, IFNγ, TNFα, IL12, IL15), then harvested and assessed by flow cytometric analysis for survival markers such as Annexin V and ToPro3. The percentage of Annexin V−/ToPro3− EOs is indicated for one representative donor out of 30 analyzed. (B) EOs were cultured in the absence or in the presence of cytokines (IL5, GM-CSF, IFNγ, TNFα, IL12, IL15), then harvested and assessed by flow cytometric analysis for the expression of CD69 surface molecules by gating on Annexin V−/ToPro3− cells. The bars indicate the percentage of CD69+ EOs. The average of 20 independent experiments is shown (% ±SD). *P < 0.05; **P < 0.01. P value was obtained by comparing the conditions in the presence of the different cytokines with the condition in the absence of cytokines (CTR). [file image_2.tif]
